# *PpMYB36* Encodes a MYB-Type Transcription Factor That Is Involved in Russet Skin Coloration in Pear (*Pyrus pyrifolia*)

**DOI:** 10.3389/fpls.2021.776816

**Published:** 2021-11-08

**Authors:** Changqing Ma, Xu Wang, Mengyuan Yu, Xiaodong Zheng, Zhijuan Sun, Xiaoli Liu, Yike Tian, Caihong Wang

**Affiliations:** ^1^College of Horticulture, Qingdao Agricultural University, Qingdao, China; ^2^Engineering Laboratory of Genetic Improvement of Horticultural Crops of Shandong Province, Qingdao, China; ^3^College of Life Science, Qingdao Agricultural University, Qingdao, China

**Keywords:** *Pyrus pyrifolia*, russet skin, lignin biosynthesis, SSR markers, *PpMYB36*

## Abstract

Fruit color is one of the most important external qualities of pear (*Pyrus pyrifolia*) fruits. However, the mechanisms that control russet skin coloration in pear have not been well characterized. Here, we explored the molecular mechanisms that determine the russet skin trait in pear using the F_1_ population derived from a cross between russet skin (‘Niitaka’) and non-russet skin (‘Dangshansu’) cultivars. Pigment measurements indicated that the lignin content in the skin of the russet pear fruits was greater than that in the non-russet pear skin. Genetic analysis revealed that the phenotype of the russet skin pear is associated with an allele of the *PpRus* gene. Using bulked segregant analysis combined with the genome sequencing (BSA-seq), we identified two simple sequence repeat (SSR) marker loci linked with the russet-colored skin trait in pear. Linkage analysis showed that the *PpRus* locus maps to the scaffold NW_008988489.1: 53297-211921 on chromosome 8 in the pear genome. In the mapped region, the expression level of *LOC103929640* was significantly increased in the russet skin pear and showed a correlation with the increase of lignin content during the ripening period. Genotyping results demonstrated that *LOC103929640* encoding the transcription factor MYB36 is the causal gene for the russet skin trait in pear. Particularly, a W-box insertion at the *PpMYB36* promoter of russet skin pears is essential for *PpMYB36-*mediated regulation of lignin accumulation and russet coloration in pear. Overall, these results show that *PpMYB36* is involved in the regulation of russet skin trait in pear.

## Introduction

Pear (*Pyrus pyrifolia*) is highly valued as a cultivated fruit crop around the world. Fruit color is one of the most vital external qualities of pear that determines market acceptance by consumers (Ma et al., 2018a). The color of pear fruit skin can be divided into two types: a ground color that includes green and yellow, and a cover color with russet (red-brown) and red colors (Heng et al., 2016). Different pear cultivars have distinct coloration, which can result from genetic or environmental factors (Heng et al., 2014). Previous studies on pear coloration have mainly explored the red skin which depends on anthocyanin biosynthesis (Wang et al., 2017; Liu et al., 2019). In addition, the russet skin is an important trait that protects pear fruits from environmental stresses caused by diseases, insects, and unfavorable weather, as well as shipping (Inoue et al., 2006). Therefore, exploring and using the genetic resources for russet skin is critical for progress in pear breeding.

Transcriptomic and proteomic approaches have been used to explore genes responsible for russet skin color in pears (Legay et al., 2015; Shi et al., 2019a). In particular, the russet skin is related to the biosynthesis of lignin, and is regulated *in vivo* by many structural and regulatory genes (Wang Y. Z. et al., 2016; Shi et al., 2019b). The structural genes that are directly involved in the biosynthesis of lignins encode phenylalanine ammonia-lyase (*PAL*), cinnamate 4-hydroxylase (*C4H*), 4-coumarate-CoA ligase (*4CL*), shikimate/quinate hydroxycinnamoyl transferase (*HCT*), coumarate 3-hydroxylase (*C3H*), cinnamoyl CoA reductase (*CCR*), cinnamyl alcohol dehydrogenase (*CAD*), caffeoyl CoA O-methyltransferase (*CCoAOMT*), and caffeic acid/5-hydroxyferulic acid O-methyltransferase (*COMT*) (Lam et al., 2017; Liu et al., 2018). Transcription factors in the R2R3-MYB family regulate the lignin biosynthesis pathway in plants (Ohtani and Demura, 2019; Geng et al., 2020). AtMYBs specifically activate lignin biosynthesis genes, which control defense-induced lignification and basal immunity in *Arabidopsis thaliana* (Chezem et al., 2017). CsMYBs were also found to regulate fruit juice sac lignification through fine-tuning of the expression of *Cs4CL* in orange (*Citrus sinensis*) (Jia et al., 2018). However, the contribution of MYB family members to russet skin coloration in pear is unclear.

In many horticultural fruit species, marker-assisted selection (MAS) for major agricultural traits has been developed. For example, molecular markers associated with pear scab resistance, harvest time, and dwarf tree architecture have been developed and applied to pear breeding programs (Terakami et al., 2006; Yamamoto et al., 2014; Wang C. H. et al., 2016). In our previous study, the gene that determines fruit russet skin was localized to linkage group 8 (LG8) of the pear consensus genetic map using simple sequence repeat (SSR) markers (Song et al., 2010). However, no candidate genes have been identified that control the pear russet skin trait on LG8. Rapid advances in DNA sequencing of the pear genome and pear haploid cell genotyping technology have provided invaluable new resources for genetics and biological research (Wu et al., 2013; Shi et al., 2019c). Recently, bulked segregant analysis combined with genome sequencing, known as BSA-seq, has proven successful for rapidly mapping genes in several vegetable and fruit species (Huo et al., 2016; Dougherty et al., 2018). Using BSA-seq, the *ABA1/ZEP* gene for thermal tolerance was efficiently identified in lettuce (*Lactuca sativa*), four major genome-wide quantitative trait loci responsible for fruit acidity were mapped on chromosomes 8 and 16 of apple (*Malus domestica*), and *CcPRR2* (*PSEUDO-RESPONSE REGULATOR 2*) was identified as a candidate gene for the control of fruit color in pepper (*Capsicum chinense*) (Huo et al., 2016; Jia et al., 2018; Lee et al., 2020).

In the present study, we aimed to elucidate the molecular mechanisms that determine the russet skin trait in pear. SSR markers were identified and mapped in the pear genome based on gene location using BSA-seq analysis. This research enhances our understanding of the molecular mechanisms underpinning russet skin coloration in pear.

## Materials and Methods

### Plant Materials

An F_1_ population of 150 individuals was derived from crossing ‘Niitaka’ (NTK, russet skin pear cultivar) with ‘Dangshansu’ (DSS, non-russet skin pear cultivar). The trees were 17 years old and were planted at a density of 2.5 × 0.5 m at the Fruit Research Station of Qingdao Agricultural University (Laiyang, Shandong Province, China). Ripening fruit samples were collected at 25, 50, 75, 100, and 125 days after full bloom (DAFB) for lignin and chlorophyll measurements and gene expression analysis. Each sample consisted of 12 fruits, and three biological replicates were harvested per time point. Young leaf samples were collected from each tree in the spring. Fruit peel was collected with a peeler, immediately frozen in liquid nitrogen, and stored at −80°C prior to its use in the experiments.

Mature ‘Korla’ pear fruits were used for infection of transgenic analysis according to Bai et al. (2019). Pear calli were induced from the flesh of young ‘Clapp’s Favorite’ (*P. communis*) fruits on NN69 (NITSCH and NITSCH 1969) solid medium. The first-generation calli were subcultured several times, and the rapidly growing soft calli were screened and maintained on Murashige-Skoog (MS) solid medium in the dark according to the protocol of Bai et al. (2019). *Nicotiana benthamiana* plants were grown *in vitro* at 25°C on solid MS medium (Murashige and Skoog, 1962).

### Chlorophyll and Lignin Measurements

Measurement of total chlorophyll content was performed as described previously (Lichtenthaler and Wellburn, 1983). In brief, pear skin tissue (0.5 g) was homogenized in 5 mL of 80% acetone and then left in the dark for 24 h. After centrifugation for 20 min at 13,000 × g, the absorbance of the supernatant was measured at 663 and 645 nm using a UV-2550 ultraviolet spectrophotometer (Shimadzu Corp., Kyoto, Japan). The chlorophyll concentration was calculated according to the protocol of Ma et al. (2018a). Total lignin was extracted from pear skin using the Lignin Content Determination Kit (Geruisi, Suzhou, Jiangsu Province, China). Three independent biological replicates were performed for each experiment.

### DNA Extraction and BSA-Seq

Leaf tissues (0.5 g each) of F_1_ plants with extreme phenotypic traits (russet or non-russet fruit skin) were ground to a powder in liquid nitrogen. DNA was isolated using the cetyltrimethylammonium bromide (CTAB) method (Ma et al., 2018a). DNA quality and concentration were assessed by electrophoresis on a 1% (w/v) agarose gel and an ultra-micro spectrophotometer (Thermo Fisher, Wilmington, DE, United States), respectively. A total of 50 individual plants, 25 with russet skin and 25 with non-russet skin, were chosen from the NTK × DSS F_1_ population for BSA-seq analysis. Two pools of genomic DNA from plants with the two extreme fruit skin phenotypic coloration traits were used to construct the segregant bulks. Each parental or bulked DNA sample was sequenced to > 30 × genome size depth using a paired-end 150 base strategy (Illumina × 10, Illumina).^1^ After quality filtering, clean reads were mapped to the pear genome^2^ using Burrows-Wheeler alignment software (Li and Durbin, 2009). SAMtools was used to obtain the read depth of the genome (Li et al., 2009). A modified G’ value method was used for the statistical analysis of allelic variations between the two bulks (Magwene et al., 2011). Venn diagrams of variants identified in different samples were constructed according to Imerovski et al. (2019). Kyoto Encyclopedia of Genes and Genomes (KEGG) pathway enrichment analysis was used to identify biological processes and functions enriched for genes with variants (Xu et al., 2021).

### Simple Sequence Repeat Marker Detection

Based on the BSA-seq results, SSRs were screened using the SSRIT website.^3^ Primers for SSR markers were designed with Primer premier 5.0 (PREMIER Biosoft International, Inc., Palo Alto, CA, United States) (Supplementary Table 3). Polymerase chain reaction (PCR) amplification assays were performed in 20 μL volumes containing 60 ng of genomic DNA, 10 μL 2 × Taq Plus Master Mix II (Vazyme, Nanjing, Jiangsu Province, China), and 0.25 μM SSR primer. Reactions were subjected to an initial denaturation at 95°C for 5 min, with 35 cycles of 94°C for 30 s, 58°C for 30 s, and 72°C for 60 s followed by a final extension step at 72°C for 10 min. Amplifications were performed in a T100^TM^ Thermal Cycler (Bio-Rad Laboratories, Hercules, CA, United States). The amplified products were separated by electrophoresis on 3.5% (w/v) agarose gels.

### Linkage Map Construction

The segregation of SSR marker loci associated with the russet skin phenotype in pear were analyzed in the 150 F_1_ offspring of the NTK × DSS cross. Genetic distances between each of the marker loci and the russet skin locus were calculated. JoinMap 4.0^4^ with the Kosambi mapping function was used for linkage analysis.

### Quantitative Real-Time PCR Analysis

Total RNA was extracted from pear tissues as described previously (Ma et al., 2018b). First-strand cDNA was synthesized using the PrimeScript^TM^ RT reagent kit (Takara, Dalian, Liaoning Province, China). LightCycler^®^ 480 SYBR Green Master (Roche, Mannheim, Germany) was used for the qRT-PCR assays with the LightCycler^®^ 480 II system (Roche, Rotkreuz, Switzerland). The *Actin* gene (GenBank: AB190176) was used as the internal control for gene expression normalization. Gene-specific primers were designed using Primer 5 (Supplementary Table 5). Data were analyzed using the 2^–ΔΔCT^ method (Livak and Schmittgen, 2001).

### PpMYB36 Genomic Sequence Cloning

The names and sequences of primers used for amplification of the *PpMYB36* genomic sequence are given in Supplementary Table 5. DNA fragments were amplified using high-fidelity DNA polymerase (Takara) using reaction conditions recommended by the manufacturer. The PCR products were purified and cloned into the PMD 19-T vector (Takara). Nucleotide sequences of 10 independent clones of each fragment per sample were determined. BLAST analysis of the amino acid sequence of PpMYB36 was performed in the *Arabidopsis* information resource,^5^ and a phylogenetic tree was constructed using MEGA 5.2 (Tamura et al., 2011). Promoter sequence analysis was performed using the PlantCARE online database.^6^

### PpMYB36 Subcellular Localization

To determine the subcellular localization of the PpMYB36 promoter, the *PpMYB36* coding region without the stop codon was subcloned into the *pMDC83* vector to generate a *35S::PpMYB36-GFP* fusion plasmid. The plasmid was then introduced into *Agrobacterium tumefaciens* strain GV3101. The leaves of 5-week-old *N. benthamiana* plants were infiltrated with GV3101 harboring *35S::PpMYB36-GFP* or mCherry (control). Subcellular localization was observed with a laser confocal microscope (×40) (FV10-ASW, Olympus, Tokyo, Japan) 3 days after transformation (Zhang et al., 2019).

### Vector Construction and Transformation

The coding sequence of *PpMYB36* was cloned and ligated into the *pBI121* vector in the sense and anti-sense directions to generate *35S::PpMYB36* and *35S::anti-PpMYB36*, respectively. The primers used are given in Supplementary Table 6. The *35S::PpMYB36* and *35S::anti-PpMYB36* plasmids were transformed separately into *A. tumefaciens* strain EHA105, and the plasmid-bearing strains were then infiltrated into fruit skin using a needleless syringe. The agro-infiltrated samples were incubated overnight in the dark at room temperature, then exposed to white light (540 μmol⋅m^–2^⋅s^–1^) with a 16 h photoperiod at 25°C in a growth chamber as previously described (Ma et al., 2019).

### *PpMYB36* Promoter Activity Assay

β-Glucuronidase (GUS) and luciferase (LUC) assays were performed as previously described (Zhao et al., 2016). *A. tumefaciens* strain GV3101 cultures harboring the *PpMYB36* promoter from russet skin pear (*ProR*) and non-russet skin pear (*ProNR*) together with the *Super* empty vector (*pCAMBIA1300*) were co-infiltrated into pear calli. *Super::LUC* was added as an internal control. GUS and LUC activities were quantified after 3 days, and the GUS/LUC ratio was used for the final quantification of the relative GUS activity. To further analyze the relative activities of the different promoters on *PpMYB36* expression, *ProR::PpMYB36* and *ProNR::PpMYB36* were cloned into *pBI121* to replace *35S::GUS*. *A. tumefaciens* GV3101 harboring *ProR::PpMYB36* and *ProNR::PpMYB36* were transiently transformed into pear skin.

### Statistical Analysis

All experiments were repeated three times. SPSS 22.0 (IBM Corp., Armonk, NY, United States) was used to conduct an analysis of variance (ANOVA) followed by Fisher’s least significant difference or Student’s *t*-test analysis. Statistically significant differences were tested by Tukey’s *post hoc* tests (*p* < 0.05).

## Results

### Phenotypic Evaluation and Color Development in Fruit Skins of F_1_ Pear Plants

**FIGURE 1:**
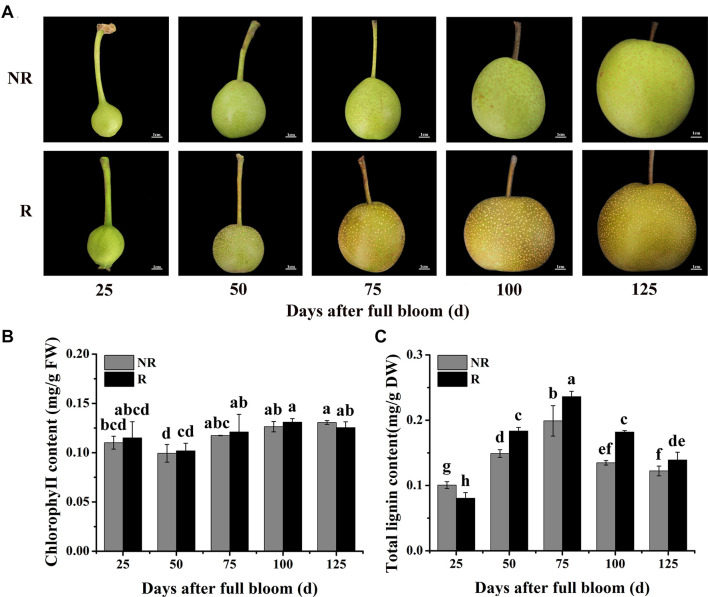
Changes in skin color and pigment contents in F_1_ plants from the cross between ‘Niitaka’ (russet skin pear cultivar, NTK) and ‘Dangshansu’ (non-russet skin pear cultivar, DSS). **(A)** Color development in pear skin of F_1_ progeny from 25 to 125 days after full bloom. **(B)** Chlorophyll and **(C)** lignin contents in pear skin of F_1_ progeny from 25 to 125 days after full bloom. NR: non-russet skin fruits; R: russet skin fruits. Different lowercase letters above bars mean significant differences between NR and R groups by Tukey’s multiple range tests (*p* < 0.05).

Pear fruits expressing the russet and non-russet skin phenotypes segregated in the F_1_ population obtained from the NTK × DSS cross. The russet skin fruits gradually turned russet in color after full bloom, whereas the non-russet skin fruits did not change color (Figure 1A). Among the 150 F_1_ progeny, there were 78 russet and 72 non-russet skin individuals. Based on a chi-square test (χ^2^ = 0.24), the segregation of the phenotypes fitted a 1:1 ratio (*p* > 0.05), indicating the pattern of genetic inheritance of this quality trait.

Furthermore, the lignin and chlorophyll contents in the skins of the two types of fruits from the F_1_ plants (russet and non-russet) after full bloom were measured. The lignin contents in the russet skin fruits increased and were significantly higher than those in the non-russet skin fruits (Figure 1B). At 75 DAFB, the lignin content in the russet skin fruits was 0.24 mg⋅g^–1^, which was approximately 1.20-fold higher than that in the non-russet skin fruits. In addition, mRNA levels of nine structural genes involved in lignin biosynthesis, *PpPAL*, *PpC4H*, *Pp4CL*, *PpCCR*, *PpCAD*, *PpbHCT*, *PpC3H*, *PpCCoAOMT*, and *PpCOMT*, were basically higher in the russet skin fruits than in the non-russet skin fruits during the ripening period (Supplementary Figure 1). However, the chlorophyll contents did not significantly differ between the russet- and non-russet skin fruits (Figure 1C). These results suggest that the enhanced russet pigmentation in pear skin can be attributed to lignin accumulation.

### Locating the Major Scaffolds to Linkage Groups Using BSA-Seq Analysis

**FIGURE 2:**
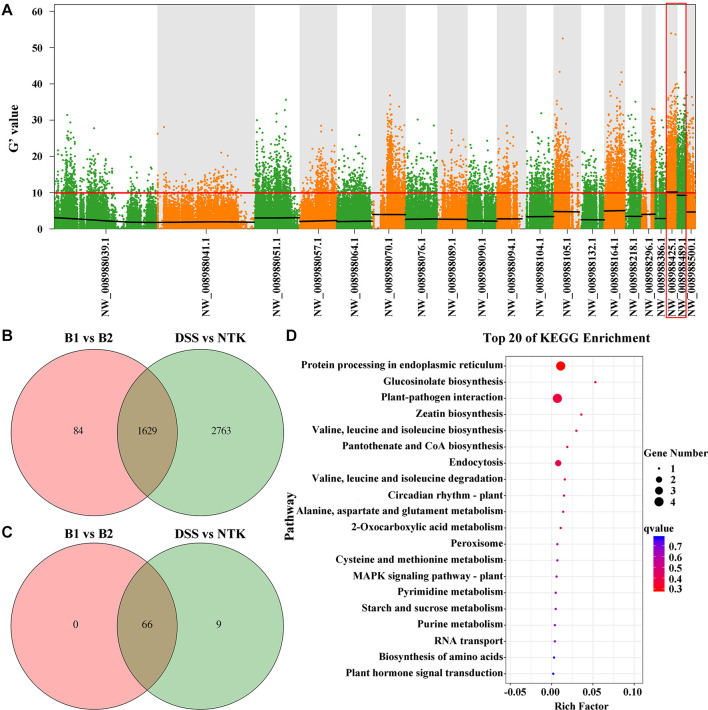
Russet skin trait locus and variant genes identified by bulked segregant analysis combined with the genome sequencing analysis. **(A)** Using the G’ algorithm to map the *PpRus* gene locus. The distribution of G’ values on the chromosome is shown. The abscissa gives the chromosome scaffold names. The colored dots represent the G’ values at each single nucleotide polymorphism locus. The red line represents the threshold of significant association. The higher the G’ value, the better is the correlation. **(B)** Venn diagram showing the intersection of all variant genes identified in pairwise analyses (B1 vs. B2 and DSS vs. NTK). DSS: ‘Dangshansu’ (non-russet skin pear cultivar); NTK: ‘Niitaka’ (russet skin pear cultivar); B1: non-russet skin fruit bulk; B2: russet skin fruit bulk. **(C)** Venn diagram representation of variant genes from a pairwise comparison of the two scaffolds NW_008988425.1 and NW_008988489.1. **(D)** KEGG pathway enrichment analysis of variant genes in the scaffolds NW_008988425.1 and NW_008988489.1.

After trimming and adapter removal, 352,649,438 paired-end clean reads from Illumina high-throughput sequencing were mapped to the pear genome (Supplementary Table 1). Small variant calling for the datasets and subsequent variant filtering generated 3, 071,265, 3,591,714, 4,337,289, and 4,163,476 variants (SNPs and Indels) for DSS, NTK, the non-russet skin fruit bulk (B1), and the russet skin fruit bulk (B2), respectively, that were uniformly distributed throughout the genome (Supplementary Table 2). G’ value association algorithms mapped the locus to the NW_008988425.1 and NW_008988489.1 scaffolds (Figure 2A), both of which are located on pear chromosome 8. Venn diagrams of all variants in the skin samples of the B1 vs. B2 and DSS vs. NTK comparisons are shown in Figure 2B. Sixty-six variant genes in the intersection of the Venn diagram in NW_008988425.1 and NW_008988489.1 were also identified (Figure 2C). The significantly enriched KEGG pathways were related to protein processing in the endoplasmic reticulum, glucosinolate biosynthesis, and plant-pathogen interaction (Figure 2D).

### Simple Sequence Repeat Markers and Genetic Linkage Map of the *PpRus* Locus

**FIGURE 3:**
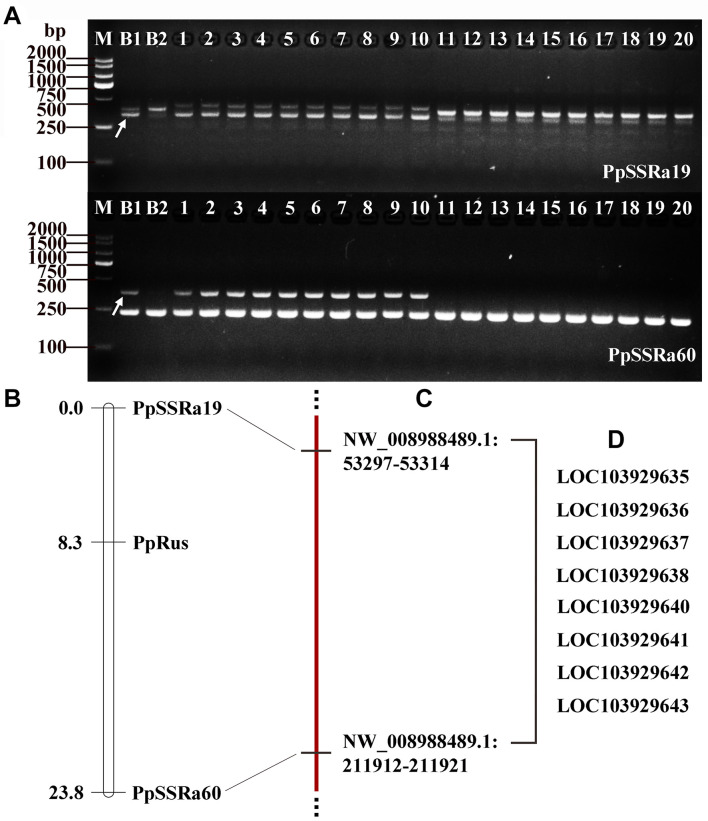
Simple sequence repeat (SSR) markers and genetic linkage mapping of the *PpRus* locus. **(A)** Two SSR markers co-segregate with *PpRus.* M: DNA size marker DL2000; B1: non-russet skin fruit bulk; B2: russet skin fruit bulk; 1–10: non-russet skin individuals; 11–20: russet skin individuals. The polymorphic DNA fragments are indicated by arrows. **(B)** Genetic linkage map around the *PpRus* locus created from a segregating F_1_ progeny population of 150 plants from the cross between ‘Niitaka’ (russet skin pear cultivar) and ‘Dangshansu’ (non-russet skin pear cultivar). **(C)** The physical positions of SSR marker loci linked to the *PpRus* locus in the pear genome. **(D)** The eight predicted genes within the region flanked by the two marker loci that are the most tightly linked to *PpRus*.

On the basis of the BSA-seq results, approximately 50 SSR primer pairs designed from sequences of NW_008988425.1 and NW_008988489.1 were analyzed (Supplementary Table 3). By screening these primer pairs on the F_1_ progeny (150 plants) from the NTK × DSS cross, two SSR markers (PpSSRa19 and PpSSRa60) were found to be linked to the *PpRus* locus. The amplification profiles of PpSSRa19 and PpSSRa60 are shown in Figure 3A. Linkage analysis revealed that the *PpRus* locus is flanked by the PpSSRa19 and PpSSRa60 loci, both of which were the nearest marker loci to the *PpRus* locus, with genetic distances of 8.3 and 15.5 cM, respectively (Figure 3B). Both of these marker loci flanking *PpRus* were located on the same scaffold (NW_008988489.1), which means that *PpRus* maps to scaffold NW_008988489.1 (53297-211921) in the pear genome (Figure 3C). There were eight genes (*LOC103929635*, *LOC103929636*, *LOC103929637*, *LOC103929638*, *LOC103929640*, *LOC103929641*, *LOC103929642*, and *LOC103929643*) among the 66 variant genes (Figure 2C) identified within the region between the marker loci PpSSRa19 and PpSSRa60 (Figure 3D).

### Expression Patterns of the Candidate Genes for Russet Skin Color in Pear Fruits

**FIGURE 4:**
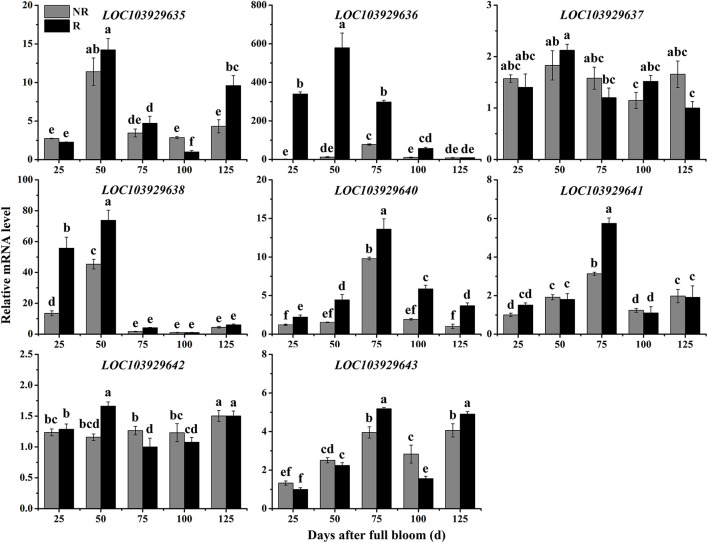
Relative expression levels of *LOC103929635*, *LOC103929636*, *LOC103929637*, *LOC103929638*, *LOC103929640*, *LOC103929641*, *LOC103929642*, and *LOC103929643* in fruit skins of F_1_ progeny from the cross between ‘Niitaka’ (russet skin pear cultivar, NTK) and ‘Dangshansu’ (non-russet skin pear cultivar, DSS). NR: non-russet skin fruits; R: russet skin fruits. Different lowercase letters above bars mean significant differences between NR and R groups by Tukey’s multiple range tests (*p* < 0.05).

To determine whether the predicted genes are involved in the trait differences between russet and non-russet skinned fruits, we quantified the expression of the eight candidate genes (Figure 3D) by qRT-PCR. The overall trends of gene transcription in the russet skin fruits showed that the genes were up-regulated (Figure 4). The *LOC103929635*, *LOC103929636*, *LOC103929637*, *LOC103929638*, and *LOC103929642* transcript levels in the russet skin fruits reached peak levels at 50 DAFB, and were 1.25-, 47.1-, 1.16-, and 1.43-fold higher, respectively, compared to the genes in the non-russet skin fruits. Additionally, the expression of *LOC103929640*, *LOC103929641*, and *LOC103929643* reached their highest levels at 75 DAFB, and were higher by 1.39-, 1.84-, and 1.31-fold, respectively, in the russet skin fruits compared to the non-russet skin fruits. Interestingly, the *LOC103929640* transcript levels followed almost the same trend as the lignin contents in the russet- and non-russet fruit skins (Figures 1C, 4).

### LOC103929640 Is Orthologous to the AtMYB36 Transcription Factor From *Arabidopsis thaliana*

**FIGURE 5:**
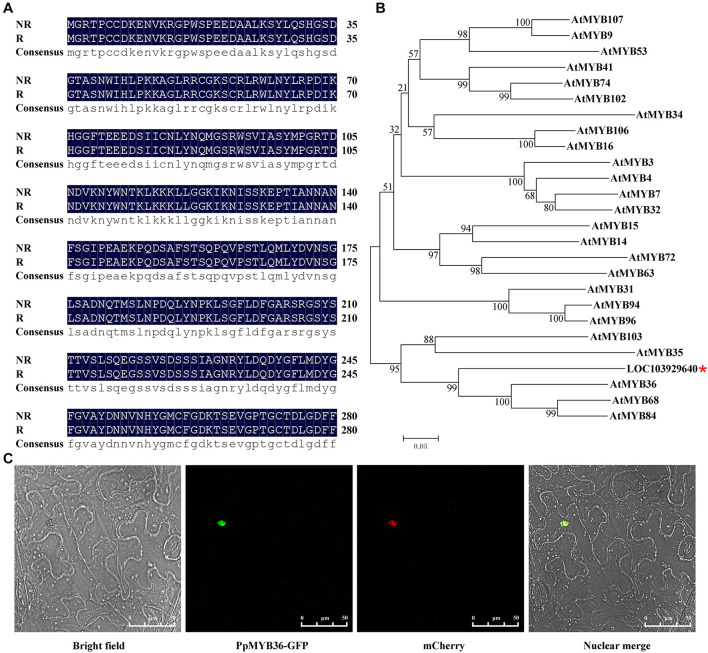
Amino acid sequences, phylogenetic tree, and subcellular localization of PpMYB36. **(A)** Amino acid sequence alignment of the two PpMYB36 proteins from russet (R) and non-russet (NR) pears. **(B)** A phylogenetic tree showing the relationships between PpMYB36 from pear and 25 MYB protein sequences from *Arabidopsis thaliana*. Bootstrap values indicate the confidence of each branch. *indicated the position of PpMYB36. **(C)** Subcellular localization of the PpMYB36-GFP fusion driven by the *CaMV35S* promoter following transient expression in leaf cells of *Nicotiana benthamiana*.

The coding sequences of *LOC103929640* were cloned from both russet and non-russet skin pears. There were no differences in the amino acid sequences of the LOC103929640 proteins between the two different fruit types (Figure 5A). To further analyze the function of LOC103929640, we performed BLAST analysis using the amino acid sequence of LOC103929640 as a search query. The result showed that LOC103929640 belongs to a MYB domain-containing protein family. Moreover, to determine the similarity and relationship of the LOC103929640 sequence to the MYBs of *Arabidopsis*, we constructed a phylogenetic tree based on an alignment of the amino acid sequences of 25 AtMYBs. The result indicated that LOC103929640 is most closely related to AtMYB36 (Figure 5B). Therefore, we named LOC103929640 PpMYB36. In addition, to determine the subcellular location of PpMYB36, we transiently expressed *PpMYB36* fused to green fluorescence protein (GFP) in *N. benthamiana* leaf cells and observed that the PpMYB36-GFP fusion protein localized to the nucleus (Figure 5C).

### Phenotypes of *PpMYB36-*Overexpressing and RNAi-Silenced Pear Fruit Skins

**FIGURE 6:**
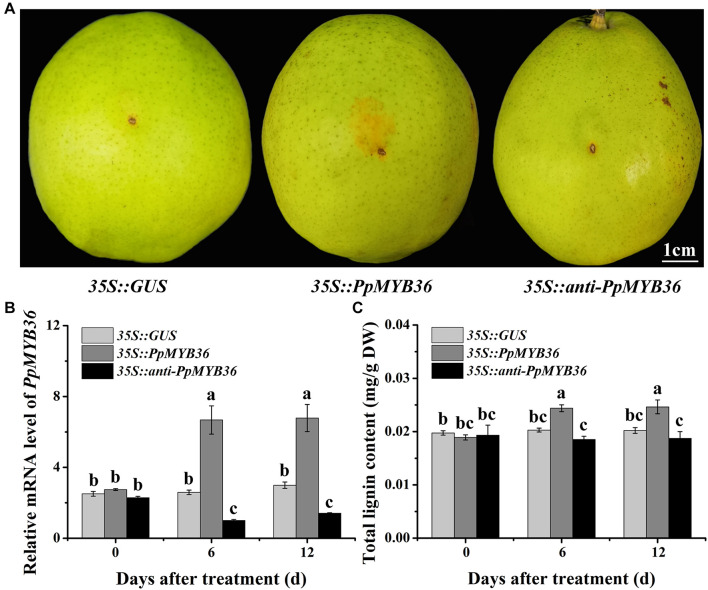
Overexpression or silencing of the *PpMYB36* gene in pear fruit skin. **(A)** Skin phenotypes of infiltrated fruits. **(B)** Relative expression levels of *PpMYB36* and **(C)** concentration of lignin in the skins of infiltrated fruits. Different lowercase letters above bars mean significant differences among the treatments by Tukey’s multiple range tests (*p* < 0.05).

To investigate the function of *PpMYB36* in the regulation of russet fruit skin coloration in pear, the constructs *35S::PpMYB36* and *35S::anti-PpMYB36* were introduced into pear skins by agroinfiltration. Fruits infiltrated with *35S::PpMYB36* displayed enhanced russet pigmentation around the injection sites, while *35S::GUS* (control) and *35S::anti-PpMYB36* fruits basically did not change color (Figure 6A). The *PpMYB36* transcription level and lignin content in pear skin expressing with *35S::PpMYB36* was significantly higher than in the control and skin infiltrated with *35S::anti-PpMYB36* (Figures 6B,C). Furthermore, the expression of nine structural genes, *PbPAL*, *PbC4H*, *Pb4CL*, *PbCCR*, *PbCAD*, *PbHCT*, *PbC3H*, *PbCCoAOMT*, and *PbCOMT*, that are involved in lignin biosynthesis, showed significantly higher levels in pear skin infiltrated with *35S::PpMYB36* compared to the control and *35S::anti-PpMYB36* infiltrated skins at 6 and 12 days after treatment (Supplementary Figure 2). These results indicate that *PpMYB36* is responsible for the increased accumulation of lignin and russet coloration in pear skin.

### Cloning and Activity Analysis of the Promoter Region of *PpMYB36*

**FIGURE 7:**
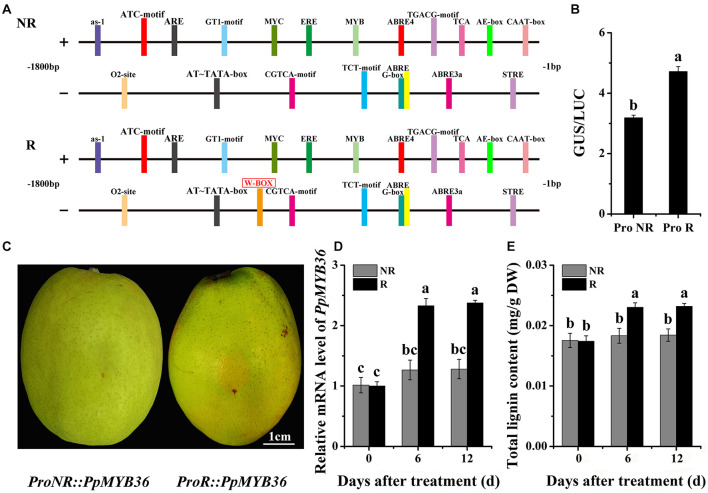
Functional analysis of the *PpMYB36* promoter region. **(A)** Diagrammatic representation of cis-elements present in the *PpMYB36* promoter (not drawn to scale). NR: non-russet skin fruits; R: russet skin fruits. **(B)** Relative GUS/LUC activity in infiltrated callus. *ProNR*: *PpMYB36* promoter from non-russet skin pear; *ProR*: *PpMYB36* promoter from russet skin pear. **(C)** Phenotypes of pear skins infiltrated with agrobacterium harboring *ProNR:PpMYB36* and *ProR:PpMYB36*. **(D)** Relative expression levels of *PpMYB36* in the skins of infiltrated fruits 0 to 12 days after agroinfiltration. **(E)** Concentration of lignin in the skins of infiltrated fruits 0 to 12 days after agroinfiltration. Different lowercase letters above bars mean significant differences between NR and R groups by Tukey’s multiple range tests (*p* < 0.05).

We characterized the *PpMYB36* upstream regions in order to determine whether the sequence polymorphisms could possibly explain the different coloration patterns in the skins of the two types of pear. Genomic DNA fragments encompassing approximately 1.8 kb of the promoter region were isolated from both russet and non-russet skin pears. We found a W-box element (−904 bp) insertion in the promoter of *PpMYB36* in russet skin pears but not in the *PpMYB36* promoter in non-russet skin pears (Figure 7A). To evaluate the relationship between the different *PpMYB36* promoter sequences and gene expression levels in russet skin and non-russet skin fruits, we first performed a dual-LUC reporter assay. *ProR* and *ProNR* were cloned into the corresponding sites of *pBI121*. LUC under the control of the *Super* promoter was the internal control for infiltration efficiency. Three days after transforming the genes into callus, the GUS and LUC activities were detected, and the GUS/LUC ratio of *ProR* was significantly higher than that of *ProNR* (Figure 7B). To test the function of the *PpMYB36* promoter, we constructed *ProR::PpMYB36* and *ProNR::PpMYB36*, which were then transiently expressed in pear skin. Fruits infiltrated with *ProR::PpMYB36* displayed enhanced russet coloration compared with fruits infiltrated with *ProNR::PpMYB36* (Figure 7C). Additionally, *PpMYB36* expression and lignin content, which were driven by *ProR*, were significantly higher than when driven by *ProNR* (Figures 7D,E). These results suggest that transcription of *PpMYB36* and lignin accumulation in the russet skin pear are influenced by the promoter sequence.

## Discussion

Russet skin is a vital trait affecting both fruit quality and stress tolerance in pear. Although several studies on russet pear have enabled a better comprehension of the mechanical causes responsible for this phenomenon (Heng et al., 2016; Shi et al., 2019b), the genetic and molecular mechanisms underpinning russet skin coloration have not been thoroughly investigated.

### Genetic Mapping of the *PpRus* Locus That Determines the Russet Skin Trait in Pear

In this study, the field phenotypes determined by visual observation of the NTK × DSS hybrids during the ripening period showed that the segregation of russet and non-russet skin fruits fitted the hypothesis of a single major gene controlling the trait. Previously, we used 121 F_1_ pear trees from the cross of ‘Whangkeumbae’ × DSS for marker screening and *PpRus* mapping; the gene locus that determines the fruit russet skin trait was localized to LG8 in pear (Song et al., 2010). Here, using 150 F_1_ pear trees from the NTK × DSS cross, we found that the *PpRus* locus was located in two scaffolds, NW_008988425.1 and NW_008988489.1 on chromosome 8 of the pear genome (Figure 2A). These results further suggest that the locus associated with the russet skin trait is located on chromosome 8 in pear. Furthermore, pear cultivar breeding is a lengthy process, largely because the trees have long juvenile stages. The MAS approach would improve the efficiency of pear breeding (Kumar et al., 2019; Fiol et al., 2021). The mapped region of the pear genome containing *PpRus*, which contains two *PpRus*-linked SSR marker loci, is useful for future pear breeding programs.

### Fine Mapping of the *PpRus* Locus and Identification of a Candidate Gene for the Pear Russet Skin Trait

Genes involved in the formation of fruit russet skin in pear are clustered into two groups: biosynthesis genes and stress-responsive genes (Legay et al., 2016). No candidate genes have been identified that control the russet skin trait on pear chromosome 8. Fine mapping of the *PpRus* locus is critical to the identification of such candidate genes. By identifying marker loci that are tightly linked to and that flank a desired gene locus, the position of the gene can be narrowed down to a small region in the chromosome or contig. Using this strategy, some candidates for the pear *PcDw* gene have been identified from the most probable region (Wang et al., 2011; Wang C. H. et al., 2016). In the present study, we predicted eight genes as candidates for *PpRus* in the region between PpSSRa19 and PpSSRa60 (Figure 3D and Supplementary Table 4). This is the first report that identifies a candidate gene possibly associated with russet skin coloration in pear. Notably, among these variant genes, *LOC103929640* (*PpMYB36*) generally exhibited an expression pattern similar to the trend in lignin contents in the two types fruits during the ripening period (Figures 1C, 4). Therefore, we suspect that *PpMYB36* plays a crucial role in the russet skin coloration phenotype in pear fruits.

### Relationship Between Lignin Accumulation and Russet Skin Coloration in Pear

Previous studies have shown that lignin biosynthesis can regulate russet skin formation in the russet skin mutant DSS of pear (Heng et al., 2016). Here, we observed considerably higher lignin contents in russet skin fruits than in non-russet skin fruits (Figure 1C), yet chlorophyll contents were not significantly different between the two fruit types (Figure 1B). Accordingly, the change in chlorophyll content may be not the main reason for russet skin coloration in pear fruits; rather, the enhanced russet pigmentation can be mainly attributed to lignin accumulation. These results are similar to observations in apple (*Malus pumila* Mill.) and grape (*Vitis vinifera*) (Legay et al., 2016; Huang et al., 2020). In addition, the crucial genes involved in lignin biosynthesis have been shown to participate in russet skin coloration in pear (Wang et al., 2014; Zhang et al., 2021). In the present study, we observed much higher expression levels of the *PpPAL*, *PpC4H*, *Pp4CL*, *PpCCR*, *PpCAD*, *PpHCT*, *PpC3H*, *PpCCoAOMT*, and *PpCOMT* genes in russet skin fruits than in non-russet skin fruits (Supplementary Figure 1), which also suggests the indispensable role of lignin accumulation in russet skin coloration in pear fruits. Hence, the mechanisms that regulate lignin biosynthesis, which in turn leads to coloration differences in pear fruit skins, require further research.

### PpMYB36 Controls the Coloration of Russet Skin Pear

As essential transcription factors, MYBs are involved in vital regulatory networks that regulate plant development, responses to biotic and abiotic stresses, and lignin biosynthesis (Dubos et al., 2010; An et al., 2019). A recent study has shown that *CsMYB36* is involved in the formation of yellow green peel in cucumber (*Cucumis sativus*) (Hao et al., 2018). In addition, *MdMYB93* regulates suberin deposition in russeted apple fruit skins (Legay et al., 2016). In the present study, russet pigmentation was enhanced while a remarkable increase in lignin accumulation was induced in pear fruits infiltrated with *35S::PpMYB36* (Figure 6). This result suggests that *PpMYB36* is crucial for regulation of lignin accumulation and russet coloration in pear. Moreover, the MYB transcription factors MYB20, MYB42, MYB43, and MYB85 are transcriptional regulators that directly activate lignin biosynthesis genes in *Arabidopsis* (Geng et al., 2020). Here, the expression levels of the nine structural genes involved in lignin biosynthesis substantially increased in pear skin infiltrated with *35S::PpMYB36*, and the expression patterns of these genes were basically consistent with *PpMYB36* transcription levels after the infiltration treatment (Figure 6B and Supplementary Figure 2). It would be interesting to investigate whether *PpMYB36* is involved in the activation of these structural genes that mediate lignin biosynthesis, thereby influencing the russet coloration of fruit skin in pear. Such work will further illustrate the regulatory role of *PpMYB36* in the russet skin trait of pear.

### Variation in the *PpMYB36* Promoter Sequence Affects Lignin Accumulation

To explore the reasons for the differential expression levels of *PpMYB36* in russet and non-russet skin pears, we compared the deduced protein sequences of PpMYB36 and found no difference between the two types fruits (Figure 5A). Nonetheless, we found a W-box (−904 bp) insertion in the promoter sequence of *PpMYB36* in the russet skin type compared to the non-russet skin type (Figure 7A; Higo et al., 1999). The results of promoter activity assays indicated that *ProR* had higher activity than *ProNR* (Figure 7B). Pear fruits infiltrated with *ProR::PpMYB36* showed enhanced russet pigmentation and also a substantial increase in lignin accumulation compared to fruits infiltrated with *ProNR::PpMYB36* (Figures 7C,E). Hence, the higher expression levels of *PpMYB36* in russet skin pears is due to the W-box insertion in the promoter region. The W-box is a WRKY protein binding element (Xie et al., 2021). Several recent studies have shown that WRKY transcription factors can bind to the promoter regions of *MYB* genes that regulate physiological and biochemical functions in plants (Lloyd et al., 2017; Liu et al., 2019). However, whether *PpWRKY* activates *PpMYB36* expression by binding to the W-box element remains to be determined. Further studies are required to reveal the mechanisms behind the activities of the different *PpMYB36* promoters and their roles in the coloration of russet fruit skin in pear.

## Conclusion

By combining BSA-seq analysis and SSR marker identification, we mapped the *PpRus* locus that determines the russet fruit skin trait in pear to the scaffold NW_008988489.1: 53297-211921 on chromosome 8 of the pear genome. Eight candidate genes were predicted in the mapped region, among which *PpMYB36* was experimentally confirmed to control russet skin coloration in pear. Moreover, a W-box (−904 bp) insertion in the *PpMYB36* promoter was found to be essential for *PpMYB36*-mediated regulation of lignin accumulation and russet coloration in pear skin. This study reveals a novel mechanism for determining russet skin coloration in pear, which is crucial for basic research and breeding applications.

## Data Availability Statement

The datasets presented in this study can be found in online repositories. The names of the repository/repositories and accession number(s) can be found in the article/Supplementary Material.

## Author Contributions

CM and CW planned and designed the research. XW, MY, XZ, ZS, XL, and YT performed the experiments, conducted the fieldwork, and analyzed the data. CM, XW, and CW wrote the manuscript. All authors contributed to the article and approved the submitted version.

## Conflict of Interest

The authors declare that the research was conducted in the absence of any commercial or financial relationships that could be construed as a potential conflict of interest.

## Publisher’s Note

All claims expressed in this article are solely those of the authors and do not necessarily represent those of their affiliated organizations, or those of the publisher, the editors and the reviewers. Any product that may be evaluated in this article, or claim that may be made by its manufacturer, is not guaranteed or endorsed by the publisher.

## Footnotes

1
https://www.illumina.com/

2
https://www.ncbi.nlm.nih.gov/assembly/GCF_000315295.1/

3
https://archive.gramene.org/db/markers/ssrtool

4
https://joinmap.software.informer.com/4.0/

5
https://www.arabidopsis.org/

6
http://bioinformatics.psb.ugent.be/webtools/plantcare/html/
