# Supplementary material for: PpMYB36 Encodes a MYB-Type Transcription Factor That Is Involved in Russet Skin Coloration in Pear (Pyrus pyrifolia)
Source: Front Plant Sci. 2021 Nov 8;12:776816. doi: 10.3389/fpls.2021.776816 (PMC8606883; doi:10.3389/fpls.2021.776816)
Supplement: Supplementary file 1 [file Data_Sheet_1.docx]

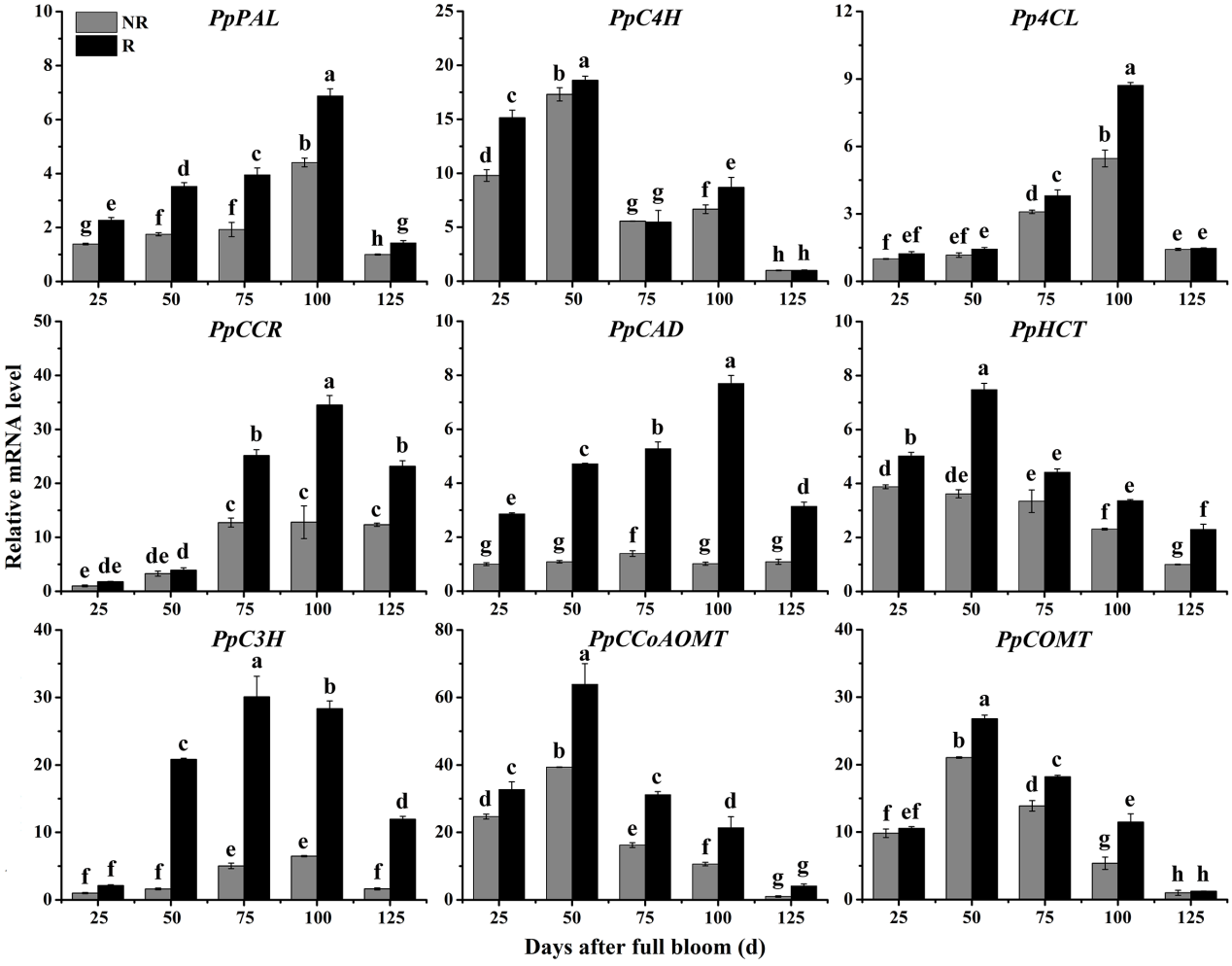


**FIGURE S1** | Relative expression of *PpPAL*, *PpC4H*, *Pp4CL*, *PpCCR*, *PpCAD*, *PpHCT*, *PpC3H*, *PpCCoAOMT*, and *PpCOMT* in the skins of russet (R) and non-russet (NR) skin fruits pear fruits from 25 to 125 days after full bloom. Different lowercase letters above bars mean significant differences between NR and R groups by Tukey’s multiple range tests (*p*<0.05).


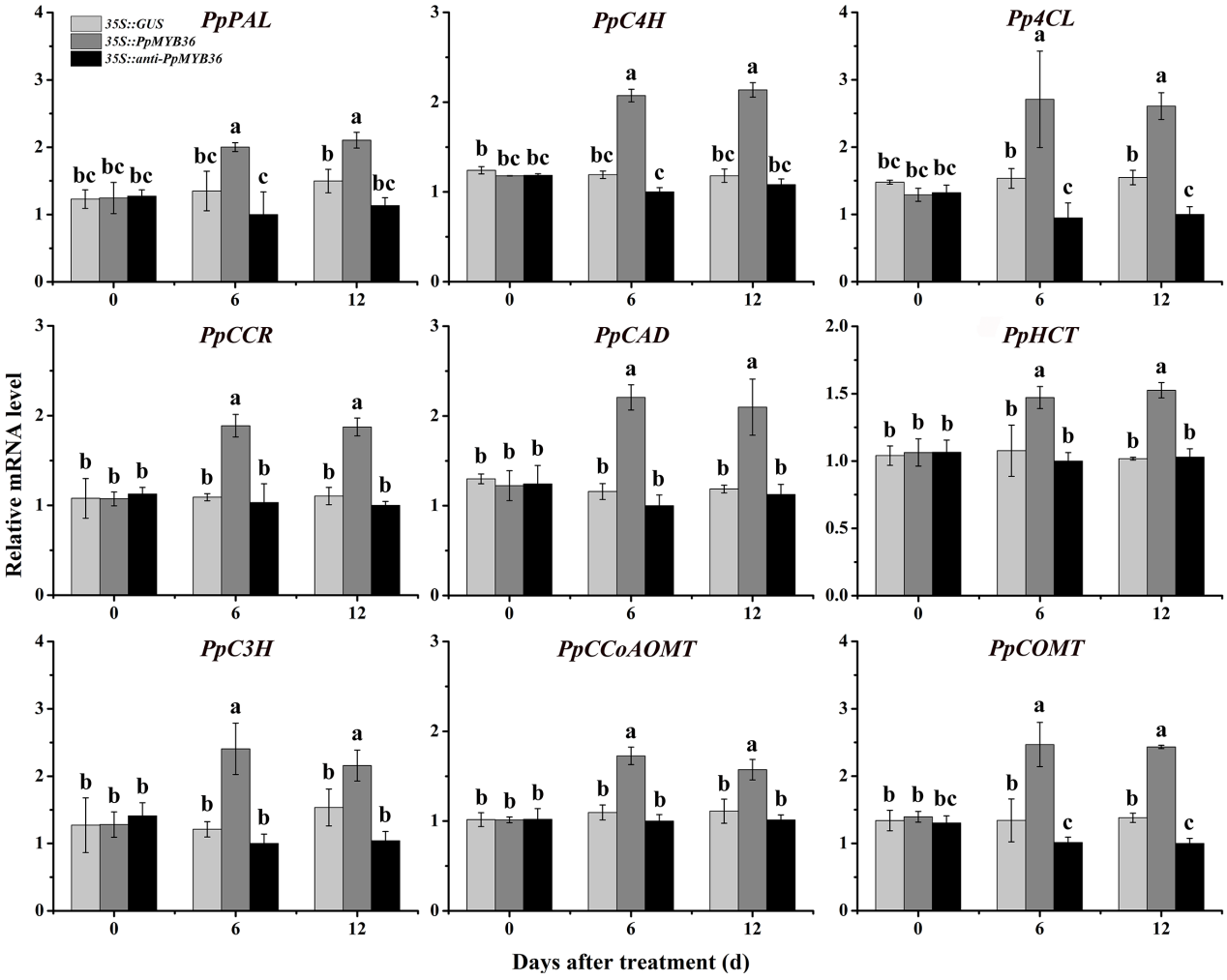


**FIGURE S2 |** Relative expression levels of genes related to lignin biosynthesis in pear fruit skins infiltrated with *35S::PpMYB36* and *35S::anti-PpMYB36* for overexpression and RNAi silencing of *PpMYB36*, respectively. The infiltration control was *35S::GUS*. Different lowercase letters above bars mean significant differences among the treatments by Tukey’s multiple range tests (*p*<0.05).

**TABLE S1** | Genome sequencing results for the parental cultivars and the two bulks.

| **Sample name** | **Clean mapped reads^1^** | **Mapping rate (%)^2^** | **Median depth** | **Coverage rate (%)^3^** |
| --- | --- | --- | --- | --- |
| DSS | 89818348 | 76.08 | 10 | 81.43 |
| NTK | 109864680 | 76.55 | 10 | 81.39 |
| B1 | 80821482 | 76.96 | 10 | 80.70 |
| B2 | 72144928 | 77.34 | 10 | 77.82 |

DSS: ‘Dangshansu’ (non-russet skin pear cultivar); NTK: ‘Niitaka’ (russet skin pear cultivar); B1: non-russet skin fruit bulk; B2: russet skin fruit bulk.

^1^Number of reads retained after trimming and adapter removal;

^2^Alignment of clean reads to the pear (*Pyrus bretschneideri*) genome (https://www.ncbi.nlm.nih.gov/assembly/GCF_000315295.1/);

^3^Coverage (≥1 read).

**TABLE S2 |** SNP-Indel statistics for the four genome sequencing samples

| **Sample name** | **SNP number** | **InDel number** | **Total** |
| --- | --- | --- | --- |
| DSS | 28,122,87 | 2,589,78 | 30,712,65 |
| NTK | 32,895,50 | 3,021,64 | 35,917,14 |
| B1 | 39,837,15 | 3,535,74 | 43,372,89 |
| B2 | 38,294,15 | 3,340,61 | 41,634,76 |

DSS: ‘Dangshansu’ (non-russet skin pear cultivar); NTK: ‘Niitaka’ (russet skin pear cultivar); B1: non-russet skin fruit bulk; B2: russet skin fruit bulk.

**TABLE S3 |** Oligonucleotide primers used for amplification of the two SSR markers linked to *PpRus* locus

| **Primer name** | **Forward primer sequence (5′–3′)** | **Reverse primer sequence (5′–3′)** | **Motif** | **No. of Repeats** |
| --- | --- | --- | --- | --- |
| PpSSRa19 | AACCAGGACACTAACTAATTTGAGG | ATTTTATTAAAGTTAAATCCAACGG | TA | 9 |
| PpSSRa60 | AAGGCAAGACAAGGCAATAATGATA | TAGGAACAAAAGCAGAGCCAAGA | TC | 5 |

**TABLE S4 |** Annotation and physical positions of eight predicted genes within the region flanked by the PpSSRa19 and PpSSRa60 marker loci on pear chromosome 8

| **Gene_ID** | **Description** | **Scafflod** | **Chr** | **Physical position (start–end)** |
| --- | --- | --- | --- | --- |
| LOC103929635 | homeobox-leucine zipper protein HAT22-like (*Pyrus bretschneideri*) | NW_008988489.1 | chr8 | 106916**–**108885 |
| LOC103929636 | homeobox protein knotted-1-like 1 (*Pyrus bretschneideri*) | NW_008988489.1 | chr8 | 118674**–**122929 |
| LOC103929637 | endo-1,4-beta-xylanase A-like (*Malus domestica*) | NW_008988489.1 | chr8 | 149046**–**154696 |
| LOC103929638 | zinc finger CCCH domain-containing protein 23-like (*Pyrus bretschneideri*) | NW_008988489.1 | chr8 | 171023**–**172475 |
| LOC103929640 | transcription factor MYB36-like (*Pyrus bretschneideri*) | NW_008988489.1 | chr8 | 183653**–**184752 |
| LOC103929641 | vacuolar protein sorting-associated protein 32 homolog 2-like (*Pyrus bretschneideri*) | NW_008988489.1 | chr8 | 188970**–**191606 |
| LOC103929642 | clathrin interactor EPSIN 1-like (*Pyrus bretschneideri*) | NW_008988489.1 | chr8 | 194240**–**199008 |
| LOC103929643 | exosome component 10 isoform X2 (*Prunus mume*) | NW_008988489.1 | chr8 | 200073**–**203926 |

**TABLE S5 |** Gene-specific oligonucleotide primers used in the quantitative real-time PCR assays.

| **Primer name** | **Sense primer (5ʹ–3ʹ)** | **Anti-sense primer (5ʹ–3ʹ)** |
| --- | --- | --- |
| *LOC103929635* | TTCTTCGTTCTCCAACTCATCTAGT | GCTCTCTGAAGGTGTCCTCTAAAG |
| *LOC103929636* | ATCAGTGTAATTTTCAGTCTCCAGG | TCCTGTCTGGCAACTGAGAGTC |
| *LOC103929637* | ACCGAGGTACAGCGACCAACTA | GTCCTTCCACTCTGGCATTCAC |
| *LOC103929638* | CACTTGACGATCACGTTATTCATAC | GAGATCGGGTTCATGGGC |
| *LOC103929640* | GGAGGAAAGATAAAGAATATCAGCA | ATGAAACAGTAGTGCTGTAACTCCC |
| *LOC103929641* | CACGATTCACAAATTAAACGATACA | AACAGTTTCTGTCGTAGCGTTTG |
| *LOC103929642* | GGAGATGGAGCAGAAGGTATTG | TGATGAGATTTGGAAAGTATGTTCT |
| *LOC103929643* | TTGGCTCCTATTCGTATAGTCACTC | TGGTGATATAGGAAGACTCAAGTGC |
| *PpPAL* | ACGCTCTCCGAACATCACCTC | GGCAAACCATTGTTGTAAAAGTCAT |
| *PpC4H* | AGATCACAGAGCCCGACGTTCATAA | AGATACCTGAAGTCGTTCCCGTTAG |
| *Pp4CL* | ATCTCTCAATGGTCTCCTTCCTCTT | GCAATAATCCCGAAGAGACAAATG |
| *PpCCR* | ATTTTTCACACCTCCTCTTTTATTG | CCAGCTATGCTCATCAATGATCT |
| *PpCAD* | AGAAAATCTGGACATACGCTGACG | CTACACCTCCAAGTCCAAGTATTCC |
| *PpHCT* | AGGGTGTTCTGTTTGTTGAGG | TCATCTGTGGAGCAGGTTGG |
| *PpC3H* | TTCTCCGCCCTATTTGCTG | CGCCGTCTTCCTCTGTATCA |
| *PpCCoAOMT* | GAGATTGGGGTCTACACTGGCTACT | ACGACCCATGATTCTTCTTATCTTC |
| *PpCOMT* | GGCTGACCACTCTACCATTACCA | TCTCCTCCAACATGCTCCACA |
| *PpActin* | TGGTGTCATGGTTGGTATGG | CAGGAGCAACACGAAGTTCA |

**TABLE S6 |** Sequences of the oligonucleotide primers used for cloning and construction of the *PpMYB36* vectors

| **Primer name** | **Sense primer (5ʹ–3ʹ)** | **Anti-sense primer (5ʹ–3ʹ)** |
| --- | --- | --- |
| *PpMYB36* | ATGGGACGCACTCCATGCT | CAAAAGAAATCACCCAAATCTGTG |
| *ProMYB36* | GATTCAAGGCAGGTATGGCTAAAGA | AAGTGAGGGAGCACAATAAGTAACC |
| *ProNR-GUS* | CATGATTACGCCAAGCTTGGTTACTTATTGTGCTCCCTCA | ACCACCCGGGGATCCTCTAGACTACAGTAAAACATCATTT |
| *ProR-GUS* | CATGATTACGCCAAGCTTGGTTACTTATTGTGCTCCCTCA | ACCACCCGGGGATCCTCTAGACTACAGTAAAACATCATTT |
| *ProNR-MYB36-pBI121* | GCACAATAAGTAACCTCTAGAATGGGACGCACTCCATGCT | ACTGACCACCCGGGGATCCTCAAAAGAAATCACCCAAATC |
| *ProR-MYB36-pBI121* | GCACAATAAGTAACCTCTAGAATGGGACGCACTCCATGCT | ACTGACCACCCGGGGATCCTCAAAAGAAATCACCCAAATC |
